# A Case-Centered Approach to Nursing Ethics Education: A Qualitative Study

**DOI:** 10.3390/ijerph17217748

**Published:** 2020-10-23

**Authors:** Won Lee, Sungkyoung Choi, Sujeong Kim, Ari Min

**Affiliations:** 1 Department of Nursing, Chung-Ang University, Seoul 06974, Korea; oness38@cau.ac.kr; 2 Department of Medical Humanities and Social Sciences, Yonsei University College of Medicine, Seoul 06974, Korea; sk0801.choi@gmail.com; 3 Department of Family Health Nursing, College of Nursing, The Catholic University of Korea, Seoul 06974, Korea; kimsu@catholic.ac.kr

**Keywords:** four topics approach, nursing ethics, nursing education, nursing student, qualitative research

## Abstract

Nurses deal with ethical decisions as they protect patients’ rights, but a consensus on effective approaches to nursing ethics education is lacking. The “four topics” method can facilitate decision-making when nurses experience ethical dilemmas in practice. This study aimed to describe nursing students’ perspectives on and experiences of a case-centered approach to nursing ethics education using the four topics method. This qualitative study consisted of two phases. First, we delivered case-centered nursing ethics education sessions to nursing students using the four topics method. Then, we conducted two focus group discussions that explored students’ perspectives on and experiences of nursing ethics education. Data were analyzed using conventional content analysis. Four themes were identified: the importance of ethics education as perceived by nursing students, problems in current nursing ethics education, the experience of case-centered nursing ethics education using the four topics approach, and suggestions for improving nursing ethics education. The case-centered approach using the four topics method is effective in enhancing nursing students’ nursing ethics ability. It is crucial to understand that nursing students would like to set up their own ethical standards and philosophy. Continuous efforts to encourage students’ participation and to provide ethical reflection opportunities during clinical practice are needed to better connect theory with clinical practice.

## 1. Introduction

Nurses have a responsibility as advocates for patients’ rights; as a consequence, nurses confront ethical quandaries daily [1,2]. As the healthcare system becomes more complex and demands for high-quality nursing care increase, nurses struggle to solve multi-faceted ethical challenges [3,4,5]. Nurses with low ethical sensitivity have difficulties making moral decisions in clinical settings [6], and thus, find it hard to advocate for patients [7,8]. More than half of U.S. nurses experience ethical dilemmas and moral distress several times a month [9], and 48% of nurses have considered leaving their position due to moral distress in a national survey conducted in New Zealand [10]. Common ethical dilemmas for nurses, for instance, includes the inability to provide the best quality care to patients due to limited resources, reduced costs, and unsafe staffing levels; carrying out physicians’ orders that a nurse considers professionally unnecessary; and the use of life support technologies to inappropriately extend life in critical care units [9,11,12]. The moral distress was found associated with low job satisfaction, burnout, and high turnover rates for nurses [1,13,14,15], thereby hindering them from providing high-quality patient care [16]. Although ethical dilemmas can create negative outcomes, ethical issues in nursing practice have received little attention.

Moral distress among nurses can decrease through training [17,18,19]. Education and training programs may be provided to enhance nurses’ ethical sensitivity and improve their understanding of decision-making processes in conflict situations [13,20,21,22,23,24,25,26]. Ethics education can enhance ethical sensitivity and awareness [21,27,28] as well as the ability to analyze ethical issues critically [29]. In particular, studies have indicated that providing ethics education to prepare nursing students is important before the newly graduated nurses enter clinical environments where ethical conflicts commonly occur [30]. Although the importance of nursing ethics education has been recognized, there is still a lack of understanding regarding it [31,32]. Courses related to ethics in the nursing education curriculum are insufficient [33], and there is a lack of educational materials [15] for nursing students. Further, there is a dearth of information regarding effective teaching methodologies to improve moral decision-making skills for nursing students [34,35,36].

Finding and applying adequate education methods to ethics regarding healthcare is a demanding task for nurse educators [37]. The four topics method, a structured framework that facilitates systematic identification of clinical ethics problems in four broad topics (medical indications, patient preferences, quality of life, and contextual features), is a way to assist ethical decision-making in healthcare settings [38]. Several studies found the method to be a useful tool in making ethical decisions in clinical settings [39,40,41]. The four topics method breaks away from the authoritative decision-making process, leading to higher-level decisions [42]; furthermore, it provides a systematic way of thinking from an ethical perspective [40]. Nurses’ better understanding of the decision-making process using the four topics method will enable them to consider a patient’s case from various ethical perspectives. Although the four topics method has been used in medical education studies, there is little information about its effectiveness in nursing ethics education. Therefore, it is necessary to evaluate this method and determine the requirements that need to be revised when applying this method to the nursing field. The purpose of this study was to describe nursing students’ perspectives on and experiences of a case-centered approach to nursing ethics using the four topics method.

## 2. Materials and Methods

### 2.1. Study Design

This is a qualitative study that aimed to explore nursing students’ experiences of ethics education using the four topics method, and we conducted it in accordance with the Consolidated Criteria for Reporting Qualitative Research (COREQ) [43]. The research team consisted of four researchers: two with a Ph.D. in Nursing and two with a Ph.D. in Public Health focusing on medical law and bioethics. All researchers had experience in qualitative research. This study consisted of two phases. First, we delivered four nursing ethics educational sessions to nursing students using the four topics method. Then, we conducted focus group discussions (FGD) that explored students’ perspectives of nursing ethics education.

### 2.2. Participants

The participants included university seniors who (1) were in their fourth year of their Bachelor of Science in nursing (BSN) program, (2) attended university in Seoul and Gyeonggi, and (3) understood the purpose of the study and agreed to participate in the study. We determined that at least one year of clinical practice experience in various clinical settings would be needed to analyze the cases provided in educational sessions. In Korea, nursing students starts their clinical practice in their third year of the BSN program; thus, we only included seniors, who are in their fourth year as participants. The exclusion criteria for the FGD were students who dropped out or were absent from the education program or did not participate in the presentation. Using convenience sampling through an online advertisement and snowball sampling, 10 nursing students from three universities in Seoul, South Korea, were recruited for this study. In July 2019, we used Facebook to recruit participants, who were asked to recommend colleagues interested in participating. All nursing students who participated in educational sessions agreed to participate in FGDs, and no one dropped out.

### 2.3. Educational Sessions

The four educational sessions lasted two hours each and took place for the 10 participating nursing students between 6 August and 21 August 2019. The course instructor was a researcher with a Ph.D. in medical law and bioethics, and the instructor did not attend the same school as the study participants. The main topics of each educational session are presented in Table 1. In the first two educational sessions, the instructor delivers lectures including ethical principles, ethical decision-making processes, issues regarding nursing ethics, and the case analysis method using four topic method. After the two lectures, students had about a week to prepare a team presentation. Students were divided into two teams (each team consisted of five students) and each team selected one case out of two possible cases: Case 1 was about an artificial abortion and Case 2 involved terminating life support for a person with brain death. Nursing students analyzed the cases themselves using the four topics method including a set of specific questions that helped identify various conditions and linked them to underlying ethical principles: beneficence, non-maleficence, autonomy, and justice [38,41]. In the remaining two educational sessions, students presented their activities and discussed their opinion. The presentation consisted of the results based on an analysis of cases, determining the legal and ethical issues involved, seeking alternatives and evaluating each alternative, and selecting alternatives and providing strategies for implementation. Students were encouraged to discuss as many alternatives as possible without judgment or criticism.

### 2.4. Data Collection

Data were collected in August 2019. Two FGDs (each team consisting of five participants) were conducted in a private meeting room. The moderator, who was educated and experienced in doctoral-level qualitative research, used a semi-structured interview guideline developed for this study. The moderator used open-ended questions and targeted probes to guide the FGD while allowing participants to talk freely about their experiences related to nursing ethics education. Interview questions were as follows: (1) Experiences regarding ethics in clinical practice: “Have you experienced any ethical situations or problems in clinical practice?” (2) Experiences about the current nursing ethics education: “What form should nursing ethics education have?”, and “Have you ever felt that nursing ethics education was insufficient while attending university or participating in clinical practice sessions? Please tell me in detail the reasons.” (3) Questions regarding a case-centered approach using the four topics: “Please tell me your experiences in this type of education, in particular using a case-centered approach utilizing the four topics,” “What is your most beneficial application of the four topics to analyze the case?”, “What was your most difficult application of the four topics to analyze the case?”, “What is different about this educational sessions compared to previous nursing ethics education?” (4) Questions regarding improvements in nursing ethics education: “What strategies could be implemented in nursing ethics education that would provide a better learning experience for nursing students?” Participants were encouraged to describe specific experiences from previous education and from the intervention sessions. The FGDs were conducted in Korean. The first FGD lasted two hours and the second one lasted two hours and 10 min. All FGDs were audio-recorded and the first author reviewed the transcripts against the audio files for accuracy.

### 2.5. Data Analysis and Trustworthiness

FGDs were transcribed verbatim in Korean, and the data were analyzed using conventional content analysis. Interview transcripts and field notes were used for data analysis. The first author extracted and coded significant words and phrases. Each student’s response in the interviews was coded appropriately. For example, the statement “I know all of the so-called ethical theories, but in reality, I don’t know how I can use those things in clinical settings” was coded to “did not know how to apply the ethical theories and principles to clinical situation.” The codes were then grouped together as subthemes and the subthemes were further grouped into overarching themes by two researchers. Subsequently, the research team reexamined the codes and engaged in multiple discussions to verify the identified subthemes and themes. We retained the words used by participants to maintain the original meanings. The quotes were selected by the research team during meetings.

To assure trustworthiness, member checking and external auditing were performed [44]. The final categories were reviewed by two nursing students who had participated in the focus group; to establish credibility (or internal consistency) of the study findings, they were asked whether their intended meaning was maintained, and they confirmed that it had been. Additionally, the study findings were reviewed by one nursing student who met the inclusion criteria but did not participate in this study, and the student answered that she mostly agreed with the study findings and had similar experiences. The finalized coding book and study findings was peer-reviewed by an external researcher who has experience in qualitative research but was not involved in the research process, to assess the accuracy (or validity) and to evaluate whether the interpretations and conclusions were supported by the study data.

### 2.6. Ethical Considerations

The study was approved by the institutional review board of Chung-Ang University (No. 1041078-201906-HRSB-192-01). Participants were informed about the study’s purpose and methods, after which they provided written informed consent. They were also informed that their participation was voluntary, and the data collected would remain confidential. Participants had the right to withdraw from the study at any time without penalty.

## 3. Results

### 3.1. General Characteristics of the Participants

A summary of participants’ demographic characteristics is provided in Table 2. Of the 10 nursing students who participated in the study, 7 were female, with a mean age of 23.0 years (±1.41). Most (8 out of 10) participants had taken ethics courses previously and identified themselves as religious.

### 3.2. Results from Focus Group Discussions

Content analysis of the data identified four main identified themes and 13 subthemes (Table 3). The four themes included: (1) the importance of ethics education as perceived by nursing students; (2) problems in current nursing ethics education; 3) the experience of case-centered nursing ethics education using the four topics method; and (4) suggestions to improve nursing ethics education.

#### 3.2.1. Theme 1: Importance of Ethics Education as Perceived by Nursing Students

Participants mentioned the importance of acquiring ethical knowledge before starting clinical practice. Without ethical knowledge, students would not even recognize ethical issues in clinical settings, let alone have the basic knowledge to judge ethical issues. One participant said, “I know what is unethical. Behaviors can vary but there seems to be a huge difference between when I know and when I don’t” (G1, P1). Most participants also noted that they encountered various ethical dilemmas and experienced moral distress during clinical practice, which created confusion regarding values. Participants resolved to make efforts to ensure that unethical judgments were not routine when they became nurses. Participants agreed that ethical awareness is critical to growth as a nursing professional. They wanted to learn about nursing ethics through discussion with others; based on their learning, they would like to create their own criteria of ethical judgments and nursing philosophy. One student said, “As specialized professionals, we have to have our own ethical principles to establish professional values. We are nursing professionals who care for people’s lives” (G2, P1).

#### 3.2.2. Theme 2: Problems in Current Nursing Ethics Education

Participants acknowledged that nursing ethics education was neglected in comparison with other subjects in the curriculum; few nursing ethics courses were offered for credit. A participant said: “credits. […] are concentrated on the subjects that can be considered as major” (G1, P1). Further, when ethics education was available, courses were structured with fixed answers, which led to difficulty in developing critical thinking. One student said, “[…] correct answers? […] as if there are fixed answers. [For ethics], it seems that such an educational method cannot cultivate students’ ability to think” (G1, P3). Given the high student-to-teacher ratio, the instructor could not facilitate all students’ discussions, and participants felt that they lacked the opportunity to think for themselves because they had to focus on passing the course. They mentioned that course contents were not memorable because students did not think and learn for themselves. Participants pointed out the lack of student motivation and voluntary participation, which made it difficult for interested students to share their thoughts in group discussion. Participants also noted that they did not know how to apply the ethical theories and principles learned in the classroom to clinical settings. Students had difficulty in recognizing and judging ethical issues in clinical practice, and they perceived a gap between theory and practice.

#### 3.2.3. Theme 3: Case-Centered Nursing Ethics Education Using the Four Topics Method

Participants acknowledged that they reviewed cases more objectively by using the four topics method. A student said that “(By using the four-topics chart) we could discuss issues within a certain framework. Summarizing and organizing analysis results were somewhat helpful” (G2, P5). However, participants mentioned that some of the questions presented in the four topics chart were difficult to apply to the given cases. Students agreed that analyzing ethical cases using the four topics method was useful because it made them think in new ways: “I found raising various questions and giving answers according to the four-topics chart to be good practice because by doing so, we could answer questions that we had never considered” (G2, P4). Through the case analysis conducted in this study, participants had an opportunity to consider others’ perspectives and ethical values. One participant said, “I believe it is good because we can know how much value each person puts in the four-topics chart” (G1, P2). Applying ethical theory and principles to cases and analyzing them using the four topics method helped them identify the values they personally find important.

#### 3.2.4. Theme 4: Suggestions to Improve Nursing Ethics Education

Participants suggested that small group education would encourage active participation, especially in a Korean cultural setting where people dislike standing out by asking questions or giving individual opinions. Participants said that the case-centered approach was helpful and suggested using cases with various ethical topics for learning several points of view. A student mentioned, “If they were real cases, I could focus better, and I could have opportunities to think about them. I wish there are some things we can remember and feel more closely” (G2, P3). They added that simple-to-complex cases need to be appropriately distributed throughout ethics education. Participants emphasized that they need opportunities to learn about ethical issues during clinical practice by sharing experiences and discussing how nurses should approach and resolve ethical problems. Through this process, students want the opportunity to think and exchange opinions on their own. Participants also stated that different evaluation methods should be used for nursing ethics education; that cultivating students’ critical thinking skills is essential; and that, particularly, students’ attitudes and participation in discussions should be included as evaluation items. One participant said, “If possible, rather than examining the students’ answers, the nursing ethics course should evaluate the level of participation in discussion and the opinions the student expresses, as well as how deeply the student participates in the course of drawing such answers and whether the student has the ability to use this tool properly” (G1, P4).

## 4. Discussion

In this qualitative study, we explored nursing students’ perspectives and experiences of ethics education to better understand current educational approaches and to present strategies for improvement. Consistent with a study by Escolar-Chua [26], our participants indicated that they encountered nursing ethics issues and moral distress during their clinical practice. Such experiences lead nursing students to imagine themselves as nurses in the future, recognize moral distress, and think about how to deal with it. Given that negative experiences in clinical practice can be impediments to staying in the nursing profession, nursing ethics education and trainings should be provided to nursing students to enhance their ethical competence, which is needed to surmount moral distress [26]. In particular, professional ethics are fundamental to the nursing profession [32], and obtaining professional competence is the most important factor in the formation of professional ethics and professionalism in students [25]. Notably, participants recognized nursing ethics education as a crucial component of professionalism and would like to create their own ethics standards and philosophy of nursing through nursing ethics education. Therefore, nurse educators need to consider providing opportunities to their nursing students for learning professional ethics and professionalism during their ethics courses.

Some issues related to current nursing ethics education need to be improved; nursing students struggled to develop critical thinking skills because the ethics education they had previously focused on lectures and did not provide enough opportunity to think for themselves and exchange their opinions with other students. In addition, nursing students had difficulties in applying theory learned in the classroom to clinical practice. To address the limitations of current nursing ethics education and to enhance students’ ability to appropriately respond to ethical concerns, it is important to develop both theory education and clinical ethics education around the ethical concerns that students may have [23]. For instance, learning activities analyzing ethical issues that students may observe or experience during their clinical practice could provide them with opportunities to apply their knowledge and skills learned from classroom education to clinical settings. Consequently, these efforts connecting theory to practice could help nursing students make ethical decisions when they enter a clinical environment after graduation. Moreover, discussion of actual situations in a systematic way can allow nursing students to realize their values and resolve ethical issues in practice [26,29]. Therefore, further work is needed to develop various ethical cases, ranging from simple to very complex, based on actual nurses’ experiences. In ethics education, the role and function of teachers can contribute to the development of professional ethics in students [25]. In Choe et al.’s study [34] on improving bioethics education, a priority was the enhancement of quality and competence in educators. Teachers should not enforce their own ethical standards and philosophy on nursing students, but should try to develop their competence through continuous education and training.

Analyzing ethical cases using the four topics method was determined to be useful by nursing students because the method guides students to analyze cases and provides direction for discussion. This finding is consistent with previous studies that established the four topics chart as a useful checklist [39]. In addition, the four topics chart made participants think in new ways. Participants mentioned that after internalizing the concepts of the four topics approach, they would be able to apply those perspectives to solve ethical issues in their work as nurses. However, nurse educators should not simply present the four topics chart as action guidelines or case analysis checklists [45], and when applying the four topics chart to nursing ethics education, some of the questions it contains should be revised considering the nursing context.

The findings of our study indicate that small group education, the case-centered approach, a connection between classroom education and clinical practice, and different evaluation methods could improve current nursing ethics education. Previous studies have also reported the use of a case-centered approach and small group discussion as the most effective ways to teach an ethics course [21,46]. For ethics development, interpersonal interactions with peers through discussion were highly effective [25]. However, there are some difficulties in small group education, such as managing the quality of discussion in small groups within a large class [21] and lack of student participation, as our participants also emphasized. Therefore, effective teaching methods for a large class [21] and strategies to motivate students’ participation should be developed to better implement the case-centered approach.

This is the first study to explore students’ experiences and perception of current nursing ethics education and case-centered nursing ethics education using the four topics method; however, it has some limitations. First, there were few participants, and they came from a limited geographic area; therefore, caution is needed in generalizing or interpreting results of this study to other nursing students. Further studies are needed to expand the target population. Second, researchers’ perceptions and intentions may result in an interpretation bias regarding participants’ responses. As mentioned in the methods section, we tried to assure trustworthiness of the study by member check, and external auditing. Third, this study examined the consequence of using the four topics method using only a qualitative method based on students’ perspectives and experiences. Therefore, future research should conduct a well-designed experimental study to identify the effects of the modified four-topic method for nursing ethics education based on our study results. Despite these limitations, this study provides useful information for the improvement of nursing ethics education.

## 5. Conclusions

Nursing students should be prepared to solve ethical issues before working in clinical settings. The study shows that a case-centered approach using the four topics method could be useful for nursing students as the structured framework of this method helps students to understand and analyze cases; this method would be helpful to train them to apply theory to specific situation. However, further research using an experimental study design that applies the modified four-topic method to the nursing field is needed to confirm our findings. Continuous efforts such as encouraging students’ active participation, using small group education, and providing ethical reflection opportunities during clinical practice will be needed to implement effective nursing ethics education that can connect the classroom and the clinic.

## Figures and Tables

**Table 1:** Description of the educational sessions.

Sessions	Topics	Learning Methods
Session 1	Introduction to course Importance of ethics in nursing Theories of ethics Principles of ethics How to utilize the four-topic method	Lecture
Session 2	Ethical decision-making process Issues in nursing ethics	Lecture
(Interim period)	(Analyze selected case using the four-topic method)	Group work
Session 3	Case study 1: Artificial abortion	Team presentation and discussion
Session 4	Case study 2: Terminating life support for a person with brain death	Team presentation and discussion

**Table 2:** Participants’ demographic characteristics.

G	P	Sex	Age	Religion	Taking Ethics Course(s)	Learning Methods in Previous Ethics Course(s)
1	1	Male	25	Catholicism	Yes	Lecture and discussion (case-based)
1	2	Female	23	Christianity	No	
1	3	Female	24	Christianity	Yes	Lecture and discussion
1	4	Male	24	Christianity	Yes	Discussion and group work
1	5	Female	22	Catholicism	Yes	Lecture and discussion (case-based)
2	1	Male	23	None	Yes	Lecture and group work
2	2	Female	23	Christianity	Yes	Lecture and group work
2	3	Female	22	None	Yes	Lecture
2	4	Female	22	Buddhism	Yes	Lecture and group work (case-based)
2	5	Female	25	Christianity	No	

Note. G = group; P = participant.

**Table 3:** Major themes and subthemes.

Theme	Subtheme
1. Importance of ethics education as perceived by nursing students	1.1 Importance of acquiring ethical knowledge before clinical practice
	1.2 Encountering ethical dilemmas and experiencing moral distress in clinical practice
	1.3 Ethical awareness that is essential to grow as a nursing professional
2. Problems of current nursing ethics education	2.1 Nursing ethics education considered to have been neglected compared to other curriculum subjects
	2.2 Barriers to participating in nursing ethics education actively
	2.3 Discrepancy between theory and practice
3. Experience of case-centered nursing ethics education using the four-topics chart	3.1 Guide to analyzing cases and providing direction for discussion
	3.2 Enables critical thinking on multi-dimensional aspects
	3.3 Opportunity to reflect on each individual’s ethical values
4. Suggestions to improve nursing ethics education	4.1 Teaching methodology that encourages active participation
	4.2 More case-centered approaches in nursing ethics education
	4.3 Opportunities for ethical reflection during clinical practice
	4.4 Different student evaluation methods in nursing ethics education
